# Mitochondrial electron transport chain functions in long-lived Ames dwarf mice

**DOI:** 10.18632/aging.100357

**Published:** 2011-08-07

**Authors:** Kashyap B. Choksi, Jonathan E. Nuss, James H. DeFord, John Papaconstantinou

**Affiliations:** ^1^ Department of Medicine, University of Texas Medical Branch, Galveston, Texas, 77555, US; ^2^ Army Research Institute of Infectious Diseases, Fort Dietrich, MD; ^3^ Department of Biochemistry & Molecular Biology, University of Texas Medical Branch, Galveston, Texas, 77555-0643

**Keywords:** Aging, Ames dwarf mice, longevity, mitochondrial function, electron transport chain activity, oxidative stress

## Abstract

The age-associated decline in tissue function has been attributed to ROS-mediated oxidative damage due to mitochondrial dysfunction. The long-lived Ames dwarf mouse exhibits resistance to oxidative stress, a physiological characteristic of longevity. It is not known, however, whether there are differences in the electron transport chain (ETC) functions in Ames tissues that are associated with their longevity. In these studies we analyzed enzyme activities of ETC complexes, CI-CV and the coupled CI-CII and CII-CIII activities of mitochondria from several tissues of young, middle aged and old Ames dwarf mice and their corresponding wild type controls to identify potential mitochondrial prolongevity functions. Our studies indicate that post-mitotic heart and skeletal muscle from Ames and wild-type mice show similar changes in ETC complex activities with aging, with the exception of complex IV. Furthermore, the kidney, a slowly proliferating tissue, shows dramatic differences in ETC functions unique to the Ames mice. Our data show that there are tissue specific mitochondrial functions that are characteristic of certain tissues of the long-lived Ames mouse. We propose that this may be a factor in the determination of extended lifespan of dwarf mice.

## INTRODUCTION

The age-associated decline in tissue function has been attributed to ROS-mediated oxidative damage due to mitochondrial dysfunction [1-5]. Mitochondrial ROS are produced by *in vivo* electron leakage from electron transport chain (ETC) complexes during normal respiration, particularly from Complex I (CI) and Complex III (CIII) [6-9]. This is consistent with the decreased capacity to produce ATP, another characteristic of aging mammalian tissues which is attributed to the selectively diminished activities of CI and CIV [10, 11] and to their vulnerability to oxidative stress [12]. On the other hand, it has been suggested that improved mitochondrial coupling and reduced release or levels of ROS production are the beneficial effects of caloric restriction that mediates longevity [13-15]. However, there is also evidence that lifespan can be increased by reduced mitochondrial ETC function in yeast, nematodes, *Drosophila* and mice [16-21]. For example, in nematodes, longevity determination is associated with an electron transport chain-mediated function that is linked to the inhibition of CIV activity within a specific tissue (intestine) and at a specific stage of development [22]. In this model the inactivated CIV in the intestine activates the mitochondrial unfolded protein response (UPR) by distal tissues. This physiological response has been proposed to be essential for lifespan extension. This raises the question of the nature of the physiological properties of decreased mitochondrial activity associated with longevity vs. the properties of dysfunctional mitochondria (ROS producing) that are associated with accelerated aging.

Several mouse models carrying specific mutations associated with increased lifespan also exhibit decreased levels of mitochondrial function and endogenous ROS, and increased resistance to oxidative stress. These models strongly support the hypothesis that ROS-mediated oxidative damage may play a key role in longevity determination. In particular, the Snell and Ames dwarf mice, which lack growth hormone (GH), thyroid stimulating hormone (TSH) and prolactin, and live ~40-60% longer than their normal littermates [23-26], have been extensively used to study the role of oxidative stress and resistance thereto in aging and longevity. These long-lived mice are also resistant to oxidative stress generated by environmental factors [23, 26-33] and their higher levels of antioxidant enzyme activities suggest that resistance to oxidative stress is a determining factor in their increased lifespan [27, 30, 31, 34]. These post-natal tissue attributes persist throughout the organism's life cycle as do those attributed to electron transport chain modulation in early stages of nematode development. These results suggest that the physiological characteristics of mitochondrial function associated with lifespan determination are established during early development and persist throughout the life cycle of the whole organism.

In this study we analyzed mitochondria isolated from several tissues of young, middle-aged and old Ames dwarf and wild-type (WT) mice to identify whether the enzyme activities of ETC complexes CI-CV and the coupled CI-CIII and CII-CIII activities exhibit unique trends in activity that may distinguish between tissues of aging or longevity models. This is consistent with the proposal that a primary effect of caloric restriction mediated extended lifespan as well as the long-lived Snell and Ames mice is due to the improvement of mitochondrial function and reduction of ROS production [13, 14, 35, 36]. We chose to examine both post-mitotic (heart and skeletal muscles) and slowly proliferating (kidney) tissues. The activities of all ETC complexes of WT and dwarf mice were compared in an attempt to identify altered functional changes associated with aging, and to identify ETC activities of specific tissues of the long-lived dwarf mice that are associated with their longevity. The aim of these studies is to determine whether ETC function in post-mitotic vs. slowly replicating tissues are discernable tissue-specific characteristics of aging *vs.* longevity.

## RESULTS

### Inhibitor-sensitive enzyme activities of heart mitochondria

**Figure 1:**
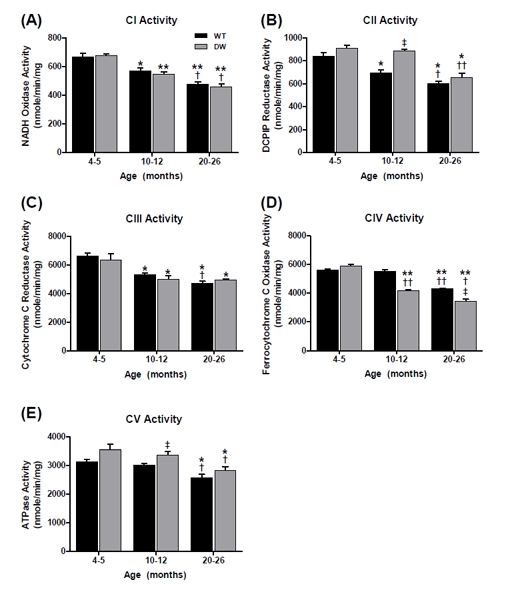
Measurement of ETC complex enzyme activities from young, middle aged, and old WT and dwarf mouse heart mitochondria Complex activities were measured spectrophotometrically as described in Methods. All activity results are the average of 4 assays from the pooled samples ± SEM for each age group. Citrate synthase activities were used to normalize mitochondrial proteins. Activities for young (4-5 months), middle-aged (10-12 months), and old (20-26 months) WT and dwarf heart CI-CV are plotted as follows: (A) CI activity. Coefficients of variance for WT and dwarf were 7.1% and 3.7% (young), 7.7% and 6.5% (middle-age), and 6.1% and 10.5% (old), respectively. (B) CII activity. Coefficients of variance for WT and dwarf were 8.3% and 6.3% (young), 7.5% and 3% (middle-age), and 6.9% and 10.5% (old), respectively. (C) CIII activity. Coefficients of variance for WT and dwarf were 6.1% and 13% (young), 3.8% and 9.5% (middle-age), and 6.2% and 3% (old), respectively. (D) CIV activity. Coefficients of variance for WT and dwarf were 1.8% and 4.6% (young), 4.3% and 3.3% (middle-age), and 2.4% and 8.7% (old), respectively. (E) CV activity. Coefficients of variance for WT were 6.1% and 10.7% (young), 3.2% and 6.6% (middle-age), and 9.3% and 8.3% (old), respectively. * - p<0.05 compared to young, ** - p<0.001 compared to young, † - p<0.05 compared to middle-aged, †† -p<0.001 compared to middle-aged, ‡ - p<0.05 compared to WT, and ‡‡ - p<0.001 compared to WT.

**Figure 2:**
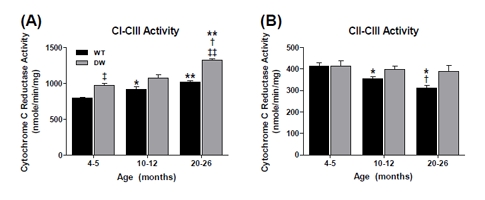
Measurement of coupled CI-CIII and CII-CIII activities from young, middle aged and old WT and dwarf mouse heart mitochondria Coupled enzyme activities were measured spectrophotometrically as described in Methods. All activity results are the average of 4 assays from the pooled samples ± SEM for each age group. Citrate synthase activities were used to normalize mitochondrial proteins. Activities for young (4-5 months), middle-aged (10-12 months), and old (20-26 months) WT and dwarf heart CI-III and CII-III are plotted as follows: (A) CI-CIII coupled activity. Coefficients of variance for WT and dwarf were 3.4% and 6.5% (young), 9% and 10.2% (middle-age), and 4.1% and 2.2% (old), respectively. (B) CII-CIII coupled activity. Coefficients of variance for WT and dwarf were 7.2% and 11.3% (young), 6.3% and 7.7% (middle-age), and 7.7% and 15.4% (old), respectively. * - p<0.05 compared to young, ** - p<0.001 compared to young, † - p<0.05 compared to middle-aged, ‡ - p<0.05 compared to WT, and ‡‡ - p<0.001 compared to WT.

To evaluate the effects of the *Prop1*^-/-^ mutation on heart muscle mitochondrial ETC function, we compared the enzyme activities of CI-CV, as well as the coupled activities of CI-III and CII-III, for young (4-5 mo), middle-aged (10-12 mo) and aged (20-26 mo) wild type (WT) and Ames dwarf mice (Figures 1 and 2). The data show that a decrease in the enzyme activities of CI-CV occurs with age and that both WT and dwarf mice exhibit this characteristic.

Rotenone-sensitive CI activity decreased by ~15% and ~19% from young to middle-age, and by old age this activity decreased by ~29% and 33% in WT and dwarf, respectively (Figure 1A). No differences were noted between the WT and dwarf CI activities at any age, thus indicating that the significant decline in CI activity is an age-associated characteristic shared by both WT and Ames mice.

Malonate-sensitive CII activity also progressively declined in WT by ~17% in middle-age and ~28% by old age (Figure 1B). In contrast, no decline occurred in dwarf CII activity from young to middle-age, and the activity decreased by ~28% in old age (Figure 1B). Thus, the decrease in CII activity is also a characteristic of aging, although the delay in the dwarf may be a characteristic of its longevity. The CII enzyme activity was also higher in dwarf mice compared to WT only at middle age (~28%).

Antimycin A (AA)-sensitive CIII activity in WT mice showed a continuous age-associated decline, *i.e.*, an ~19% decline by middle age and ~28% by old age, while dwarf mice declined by ~21% in middle age and remained at this level of activity in old age (Figure 1C). No differences were seen between WT and dwarf CIII activity at any age. These changes are thus a characteristic of aging that also occurs in the long-lived Ames mice.

KCN-sensitive CIV activity in the WT mice remained unchanged at middle age and then decreased by ~24% in old animals, while dwarf mice showed a continuous decline with aging - an ~29% decline in middle age and ~42% by old age (Figure 1D). This marked decrease in CIV activity in dwarf compared to WT mice suggests lower rates of oxygen consumption that may be a prolongevity characteristic.

Oligomycin-sensitive CV activity in both WT and dwarf mice decreased only in old age, by ~16% and ~21%, respectively (Figure 1E). In comparison, the dwarf CV activity was higher than in the WT at young and middle age (~12%), suggesting a higher level of ATP production in dwarf mice at these ages.

Overall, most ETC enzyme activities in WT and dwarf heart muscle showed a tendency towards an age-associated decline in activity, suggesting that this is a general physiological characteristic of aging shared by both WT and long-lived Ames mice. However, the only significant difference in activity occurred with the dwarf CIV at middle and old age, and involved the rate at which CIV activity declined, suggesting that these lower levels of enzyme function in the dwarf mice may be a characteristic of longevity. These results also raise the question of the nature of the physiological basis for decline of ETC enzyme activity in WT *vs.* dwarf mitochondria.

In contrast to the general trend of age-associated loss of heart muscle enzyme activity, there was an increase in CI-CIII coupled activity in both WT and dwarf mice, although the increase was significantly higher in the dwarfs (Figure 2A).Thus, the higher CI-CIII coupled activity of dwarf mice at young (~22%), middle (~17%) and old age (~30%) suggests a tighter coupling of CI-CIII associated with the longevity phenotype at all ages (Figure 2A). On the other hand, the coupled CII-III activity showed an age-associated decline in activity specific to the WT - an ~15% decline in middle age and ~25% decline by old age (Figure 2B). In contrast, no significant age-related change in coupled CII-CIII activity was seen in dwarf mice. These data suggest that the higher level of coupled CII-CIII activity, and failure of this activity to decline with age in the dwarf, may be a characteristic of the longevity phenotype.

### Inhibitor-sensitive enzyme activities of the pectoralis

**Figure 3:**
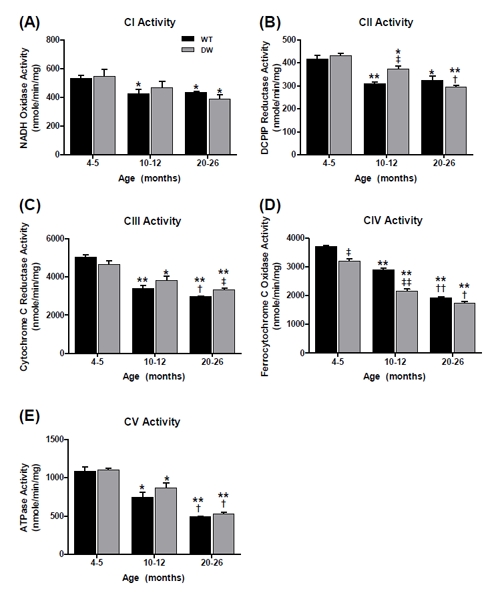
Measurement of ETC complex activities from young, middle aged and old WT and dwarf mouse pectoralis muscle mitochondria Complex enzyme activities were measured spectrophotometrically as described in Methods. All activity results are averages of 4 assays from the pooled sample ± SEM for each age group. Citrate synthase activities were used to normalize mitochondrial proteins. Activities for young (4-5 months), middle-aged (10-12 months), and old (20-26 months) WT and dwarf pectoralis CI-CV are plotted as follows: (A) CI activity. Coefficients of variance for WT and dwarf were 8.4 % and 19.3% (young), 14.5% and 19.6% (middle-age), and 4.1% and 16.2% (old), respectively. (B) CII activity. Coefficients of variance for WT and dwarf were 6.7% and 3.7% (young), 3.5% and 7.7% (middle-age), and 11.8% and 5.1% (old), respectively. (C) CIII activity. Coefficients of variance for WT and dwarf were 6% and 7.3% (young), 9.6% and 11.3% (middle-age), and 2.8% and 5.7% (old), respectively. (D) CIV activity. Coefficients of variance for WT and dwarf were 3.2% and 5% (young), 4.9% and 5.7% (middle-age), and 3.3% and 8.7% (old), respectively. (E) CV activity. Coefficients of variance for WT and dwarf were 9.9% and 4.5% (young), 18.2% and 14.1% (middle-age), and 2.2% and 7.4% (old), respectively. * - p<0.05 compared to young, ** - p<0.001 compared to young, † - p<0.05 compared to middle-aged, †† -p<0.001 compared to middle-aged, ‡ - p<0.05 compared to WT, and ‡‡ - p<0.001 compared to WT.

**Figure 4:**
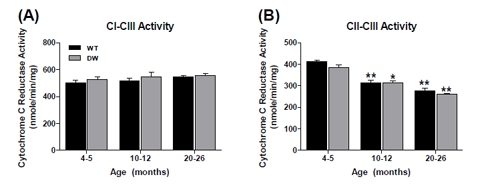
Measurement of coupled mitochondrial ETC complex activities from young, middle aged and old WT and dwarf mouse pectoralis muscle mitochondria CI-III and CII-III coupled enzyme activities were measured spectrophotometrically as described in Methods. All activity results are the average of 4 assays from the pooled samples ± SEM for each age group. Citrate synthase activities were used to normalize mitochondrial proteins. Activities for young (4-5 months), middle-aged (10-12 months), and old (20-26 months) WT and dwarf pectoralis CI-III and CII-III are plotted as follows: (A) CI-CIII coupled activity. Coefficients of variance for WT and dwarf were 8.2% and 6.1% (young), 7% and 12.9% (middle-age), and 4.7% and 5.4% (old), respectively. (B) CII-CIII coupled activity. Coefficients of variance for WT and dwarf were 2.9% and 6.3% (young), 6.2% and 5.9% (middle-age), and 10.2% and 2.9% (old), respectively. * - p<0.05 compared to young, ** - p<0.001 compared to young, and † - p<0.05 compared to middle-aged.

**Figure 5:**
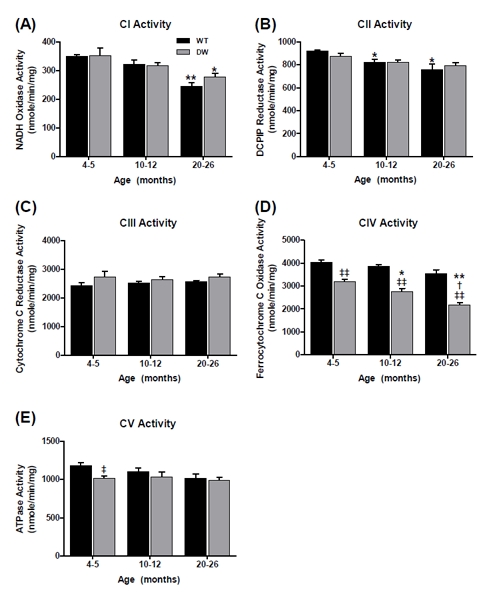
Measurement of ETC complex activities from young, middle aged and old WT and dwarf mouse quadriceps muscle mitochondria Complex enzyme activities were measured spectrophotometrically as described in Methods. All activity results are the average of 4 assays from the pooled samples ± SEM for each age group. Citrate synthase activities were used to normalize mitochondrial proteins. Activities for young (4-5 months), middle-aged (10-12 months), and old (20-26 months) WT and dwarf quadriceps ETC CI-CV are plotted as following. (A) CI activity. Coefficients of variance for WT and dwarf were 3.2 % and 15.1% (young), 9.4% and 6.3% (middle-age), and 10.5% and 9.5% (old), respectively. (B) CII activity. Coefficients of variance for WT and dwarf were 1.9% and 5.7% (young), 7.2% and 5.9% (middle-age), and 12.9% and 5.8% (old), respectively. (C) CIII activity. Coefficients of variance for WT and dwarf were 8% and 13.9% (young), 4.6% and 9.4% (middle-age), and 3.6% and 8.7% (old), respectively. (D) CIV activity. Coefficients of variance for WT and dwarfs were 5.9% and 6.9% (young), 4.6% and 9.5% (middle-age), and 10.5% and 8.7% (old), respectively. (E) CV activity. Coefficients of variance for WT and dwarfs were 6.5% and 5.1% (young), 10.2% and 13% (middle-age), and 11.6% and 8% (old), respectively. * - p<0.05 compared to young, ** - p<0.001 compared to young, † - p<0.05 compared to middle-aged, ‡ - p<0.05 compared to WT, and ‡‡ - p<0.001 compared to WT.

**Figure 6:**
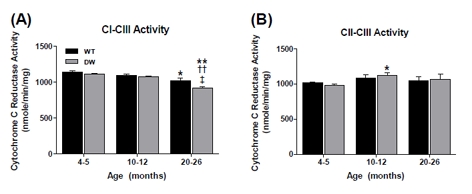
Measurement of coupled mitochondrial ETC complex activities from young, middle aged and old WT and dwarf mouse quadriceps muscle mitochondria CI-III and CII-III coupled enzyme activities were measured spectrophotometrically as described in Methods. All activity results are the average of 4 assays from the pooled samples ± SEM for each age group. Citrate synthase activities were used to normalize mitochondrial proteins. Activities for young (4-5 months), middle-aged (10-12 months), and old (20-26 months) WT and dwarf quadriceps CI-III and CII-III are plotted as follows: (A) CI-CIII coupled activity. Coefficients of variance for WT and dwarf were 3.1% and 2.5% (young), 2.8% and 2.5% (middle-age), and 6.5% and 3.1% (old), respectively. (B) CII-CIII coupled activity. Coefficients of variance for WT and dwarf were 2.2% and 4.3% (young), 9.2% and 6.8% (middle-age), and 10.5% and 13.3% (old), respectively. * - p<0.05 compared to young, ** - p<0.001 compared to young, †† - p<0.001 compared to middle-aged, and ‡ - p<0.05 compared to WT.

To evaluate the physiological effects of the *Prop1^(−/−)^* mutation on skeletal muscle mitochondrial ETC function, we chose to study the pectoralis (red), an aerobic muscle with high levels of mitochondria, and the quadriceps (white), an anaerobic muscle with lower levels of mitochondria. The pectoralis is a highly aerobic muscle consisting of slow twitch fibers (type 1), high mitochondrial content and high myoglobin levels, which improve the delivery of oxygen. We compared the enzymatic activities of the pectoralis (Figures 3, 4) and quadriceps (Figures 5, 6) ETC CI-CV as well as their CI-III and CII-III coupled activities for all three ages in both WT and dwarf mice. The data clearly show different enzyme activity profiles between WT vs. dwarf mice. All of the enzyme activities declined with age in both WT and dwarf; by old age this decline was ~19% and ~28% in CI; ~23% and ~32% in CII; ~41% and 28% in CIII; ~48% and ~46% in CIV; and ~55% and ~52% in CV, respectively (Figure 3A-E). These data are consistent with our previous studies which showed an age-associated decline in CI-CV activities in C57BL/6 male mice indicating that this is not due to genetic background [37]. There were only minor differences between the dwarf and WT in CII and CIII activities. However, in CIV, the dwarf had a lower activity at both young and middle age compared to WT (by ~13% and ~25%, respectively), though by old age their activities are similar (Figure 3D). Our data suggest that the decline in pectoralis CI-CV activities is an age-associated characteristic of both WT and dwarf pectoralis, and that the more rapid decline in activity in the dwarf may be a prolongevity characteristic (which also occurs in dwarf heart muscle).

Interestingly, the CI-CIII coupled activity showed no age-associated changes (Figure 4A), thus being unique in its resistance to aging. In previous studies we found similar resistance in the pectoralis of aged C57BL/6 mice [37]. On the other hand, there were ~33% declines in CII-CIII coupled activity in both WT and dwarf mice (Figure 4B), suggesting that this is a general characteristic of aging, not affected by longevity. Interestingly, we demonstrated in our previous studies that the CII-CIII activity of C57BL/6 mice is not affected by aging, suggesting differences that may be due to genetic background [37].

### Inhibitor-sensitive enzyme activities from quadriceps

We chose to study the mitochondrial ETC activities of the quadriceps because it consists of fast-twitch type I fibers whose physiological characteristics include fewer mitochondria. The high levels of glycolytic enzymes enable these fibers to respire anaerobically. The data in Figure 5 show that there was a significant decrease in WT CI and no effect on CII, CIII and CV activity; there was a noticeable but not quite statistically significant drop in CIV activity by old age. In the dwarf quadriceps, there was a significant decrease in CI activity and a dramatic decline in CIV activity associated with aging, i.e., ~14% decline in middle age and ~32% decline by old age (Figure 5D). Thus, as with the pectoralis the CIV activity of the dwarf quadriceps exhibited a specific sharp decrease in activity, suggesting that this is a characteristic of longevity.

With respect to the CI-CIII and CII - CIII coupled activities the data showed essentially no change in the WT and only a minor decrease in CI - CIII in the dwarf (Fig. 6A, 6B).

Overall, the two physiologically and functionally different skeletal muscles showed certain unique profiles that emphasize the differences in ETC enzyme activities with aging in both WT and dwarf mice. The difference in CIV activity of the dwarf vs. the WT was the only major difference associated with longevity.

### Inhibitor-sensitive enzyme activities from kidney

**Figure 7:**
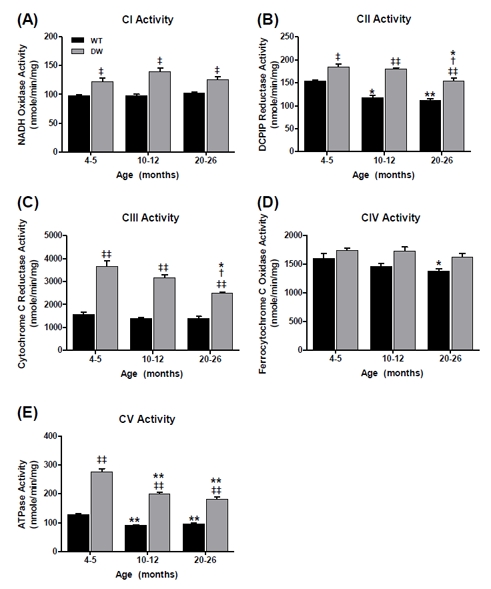
Measurement of ETC complex activities from young, middle aged and old WT and dwarf mouse kidney mitochondria Complex enzyme activities were measured spectrophotometrically as described in Methods. All activity results are the average of 4 assays from the pooled samples ± SEM for each age group. Citrate synthase activities were used to normalize mitochondrial proteins. Activities for young (4-5 months), middle-aged (10-12 months), and old (20-26 months) WT and dwarf kidney ETC CI-CV are plotted as follows: (A) CI activity. Coefficients of variance for WT and dwarf were 3.9 % and 10.8% (young), 5.6% and 9.5% (middle-age), and 4.9% and 9.2% (old), respectively. (B) CII activity. Coefficients of variance for WT and dwarf were 2.6% and 7.5% (young), 6.5% and 3.6% (middle-age), and 6.8% and 7.7% (old), respectively. (C) CIII activity. Coefficients of variance for WT and dwarf were 13.7% and 12.9% (young), 10.2% and 8.8% (middle-age), and 12.7% and 4.3% (old), respectively. (D) CIV activity. Coefficients of variance for WT and dwarf were 9.9% and 4.8% (young), 6.8% and 8% (middle-age), and 4.6% and 7.7% (old), respectively. (E) CV activity. Coefficients of variance for WT and dwarf were 5.5% and 8% (young), 3.4% and 6.1% (middle-age), and 7.5% and 9% (old), respectively. * - p<0.05 compared to young, ** - p<0.001 compared to young, † - p<0.05 compared to middle-aged, ‡ - p<0.05 compared to WT, and ‡‡ - p<0.001 compared to WT.

**Figure 8:**
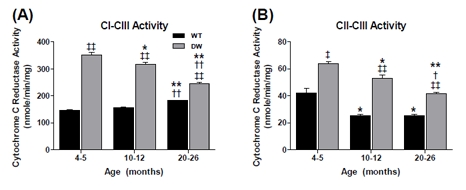
Measurement of coupled mitochondrial ETC complex activities from young, middle aged and old WT and dwarf kidney mitochondria Coupled enzyme activities were measured spectrophotometrically as described in Methods. All activity results are the average of 4 assays from the pooled samples ± SEM for each age group. Citrate synthase activities were used to normalize mitochondrial proteins. Activities for young (4-5 months), middle-aged (10-12 months), and old (20-26 months) WT and dwarf kidney CI-III and CII-III are plotted as follows: (A) CI-CIII coupled activity. Coefficients of variance for WT and dwarf were 4.1% and 5.2% (young), 4.4% and 5.6% (middle-age), and 1.5% and 3.4% (old), respectively. (B) CII-CIII coupled activity. Coefficients of variance for WT and DW were 15.8% and 5% (young), 8% and 8.7% (middle-age), and 5.4% and 5% (old), respectively. * - p<0.05 compared to young, ** - p<0.001 compared to young, † - p<0.05 compared to middle-aged, †† - p<0.001 compared to middle-aged, ‡ - p<0.05 compared to WT, and ‡‡ - p<0.001 compared to WT.

We chose to study the mitochondrial ETC activities of the kidney because its high urea levels cause high levels of endogenous oxidative stress [38]. The kidney is also prone to relatively high levels of hydroxynonenol (HNE) adduct formation in mitochondrial proteins [39]. In our previous studies we showed a direct relationship between increased protein modification and decreased enzyme function for CI, CII and CV, suggesting increased endogenous oxidative stress with aging due to mitochondrial dysfunction [40]. In the current studies we evaluated the physiological effects of the *Prop1^−/−^* mutation on kidney mitochondrial ETC function. We measured the enzymatic activities of CI-CV as well as the coupled activity of CI-III and CII-III for all three ages in WT and dwarf mouse kidneys (Figures 7 and 8). In contrast to the heart and skeletal muscles, we found major differences in CI, CII, CIII and CV enzyme activities between WT and dwarf mouse kidneys. In WT kidney, no changes were seen in CI and CIII activities (Figure 7A and 7C); and there was an age-related decline in function in WT CII (~23% in middle age and ~28% by old age, Figure 7B); in CIV (~14% by old age, Figure 7D); and CV (~29% in middle age and no further decline in old age, Figure 7E).

On the other hand, the dwarf kidney showed no age-related changes in CI and CIV activities (Figure 7A and 7D, although these activities were significantly higher than in WT), whereas the other enzyme functions (CII, CIII, CV) declined with age. In particular, CII and CIII function decreased at old age by ~17% and ~32%, respectively (Figure 7B and 7C). CV activity also showed a continuous decline with aging, such that the activity decreased by ~28% in middle age and ~35% at old age (Figure 7E).

Our data show that the overall enzyme activities of the kidney ETC complexes were significantly higher at all ages in dwarf than in WT mice. In fact, the CIII and CV activities were more than two-fold higher in dwarf at young age compared to the WT. Thus, although the WT and dwarf activities decreased progressively with aging, the significant differences between them suggest that the higher levels of activity in the dwarf may be an organ-specific characteristic of longevity.

The data in Figure 8A show an age-associated increase in WT CI-CIII coupled activity (~26%). On the other hand, the CI-CIII coupled activity in the young dwarf kidney is significantly higher than in the WT (by ~2.5 fold) and progressively declines with age; the dwarf activity remains higher at all ages. Similarly, CII-CIII coupled activity in the WT kidney, which is significantly lower than in the dwarf, declines by ~40% in middle age, but no further in old age (Figure 8B). As with the CI-CIII coupling, the CII-CIII coupled activities in dwarf kidneys declined with age; CI-CIII activity decreased by ~10% in middle and ~30% in old age (Figure 8A), and CII-CIII activity decreased by ~17% and ~35% in middle and old age, respectively (Figure 8B).

Overall, the most significant differences in enzyme activities between the dwarf and WT occurred in the kidney. These data suggest that ETC activities of tissues of aging complex organisms may exhibit tissue-specific physiological characteristics of longevity.

## DISCUSSION

The ~40-60% increased lifespan of the long-lived Ames dwarf mouse is attributed to a mutation of the Prop 1 locus that results in poor development of the anterior pituitary and a deficiency of GH, TSH and prolactin [23, 24, 26]. Numerous studies have indicated that the Ames as well as the related Snell dwarf mouse models exhibit an increased level of resistance to oxidative stress generated by extrinsic factors such as hydrogen peroxide, paraquat and 3-NPA [23, 25, 26, 29, 33, 34, 41, 42]; a recent study has identified mitochondrial ETC function(s) that may contribute to the longevity of these mice [36] as well as such factors as oxidative modifications of specific ETC complex proteins in various tissues of aging WT mice [37, 40, 43].

In a recent study, however, it has been shown that the tissue-specific knockdown of ETC-CIV by manipulation of cytochrome c oxidase-1 subunit Vb/COX4, in intestinal and neuronal tissues of nematodes, at a specific developmental stage, results in increased lifespan [22]. This study presents direct evidence that a tissue-specific reduction of mitochondrial function establishes longevity (rate of aging) for the whole organism. Interestingly, our comparison of the progression of mitochondrial ETC functions with age, e.g., enzyme activities of ETC CI-CV and the coupled electron transport activity of CI → CIII and CII → CIII in various tissues from long-lived Ames mice to corresponding tissues from WT controls, has identified mitochondrial ETC functions that occur in both aging WT and dwarf tissues, as well as potential prolongevity functions unique to the dwarf tissues. Our studies raise the question of whether a progressive developmental tissue-specific reduction of mitochondrial ETC function influences the rate of aging of the WT vs. dwarf tissues. Furthermore, the progressive tissue-specific changes in ETC function may be a mechanism for the differential rate of tissue-specific aging in higher organisms. Our studies thus raise the question of: a) the mechanism of establishing differential tissue specific ETC function and b) does the hormonal deficiency in the dwarf mice play a role in establishing ETC functions associated with longevity?

The majority of changes in heart and skeletal muscle mitochondrial activities are characteristics of aging that are shared by both the WT and long-lived mice with the exception of CIV. We thus propose that the decreased activities that occur in the WT tissues may be indicative of age-associated mitochondrial dysfunction whereas the rate of decline of CIV activity in the muscle tissues, which is faster in the dwarfs, may be indicative of a beneficial reduction of mitochondrial function influenced by factors of longevity determination. The decreased CIV activity may thus be determined by a circulating (humeral) prolongevity factor that may lower respiration without causing mitochondrial dysfunction, possibly leading to lower energy production, and resistance to oxidative stress [33, 41]. The absence or low abundance of such a factor in WT tissue would favor the development of mitochondrial dysfunction associated with the aging of WT tissues. This is consistent with the proposal that improved mitochondrial coupling and reduced ROS production are the beneficial effects of caloric restriction mediated longevity [13-15].

Our data show significant tissue-specific differences in activities between the WT and dwarf kidneys. Thus, the stabilized and elevated tissue-specific levels of CI, CII, CIII and CV activities and elevated coupled CI - CIII and CII - CIII activities are unique physiological ETC characteristics of the kidney in long-lived dwarf mice that are established and maintained throughout the post-natal dwarf life cycle. The metabolic consequences of these altered mitochondrial ETC functions may be kidney-specific physiological characteristics that maintain healthy kidney function thereby supporting an extended lifespan in these animals. Our results suggest that mitochondrial function associated with longevity determination involves an early establishment of tissue-specific ETC activities and that the mechanism of longevity determination must establish ETC functions that provide the life-long physiological needs of that tissue.

The concept of circulating factor(s) that promote ETC mediated longevity has been proposed to be a cell non-autonomous mechanism that regulates ETC-mediated longevity [22]. On the other hand such a mechanism may explain the consequences of mitochondrial dysfunction of the Klotho mutation and its overexpression [44]. For example, Klotho is a circulating humeral factor that regulates mitochondrial function (oxidative stress), and is synthesized mainly by the kidney and brain. It thus exhibits functional characteristics attributed to a circulating factor whose activity is associated with mitochondrial homeostasis and longevity. The occurrence of such a mechanism in higher organisms such as the mouse may be the basis for the developmental establishment of differential mitochondrial function but must also explain the tissue-specific variations in ETC activities in post-natal tissues. It remains to be seen if Klotho activates the mitochondrial UPR as part of its prolongevity activity.

Increased lifespan in yeast has been attributed to the deletion of the YGRO76C (AFO1) gene which encodes the mitochondrial ribosomal protein of the small subunit, mDAP-3 [45], is yet another example in which respiratory deficiency, resistance to oxidative stress and decreased levels of ROS production increases lifespan [46, 47]. One of the physiological characteristics of this long-lived mutant involves its defense against endogenous ROS (oxidative stress). This mitochondrial ribosomal protein is conserved between yeast and human cells and its functions include translation as well as apoptosis. However its possible function in aging of higher eukaryotes remains to be demonstrated. Further studies should reveal whether there are functional homologs of AF01 in higher eukaryotes and whether its tissue-specific and developmental stage-specific attenuation activates the mitochondrial misfolded protein response and lifespan extension.

Aged mammalian tissues show a decreased capacity to produce ATP, and this altered mitochondrial function has been attributed to the selectively diminished activities of CI and CIV [10]. Furthermore, CIV appears to be a particularly “vulnerable” activity in aging [11] and oxidative stress [48]. This may be due to the oxidative modifications of COX2, which is malondialdehyde-modified by ~50% in the aged kidney [40, 49]. It is interesting that the induction of the mitochondrial unfolded protein response (UPR) which is activated in response to mitochondrial perturbation is specific to the ETC longevity pathway in the CIV perturbed nematodes [22]. It is thus possible that non-damaging oxidative modification of proteins of CIV may elicit the UPR, thereby stimulating the expression of the mitochondrial associated chaperones and their protective effects.

Our studies confirm that the CI and CIV activities of WT mice decrease with age. Apparently, although these decreased activities of the dwarf heart, pectoralis and quadriceps may decrease ATP levels this does not appear to affect the longevity of the Ames mouse. We thus propose that decreased CI and CIV activity may be an age-associated mitochondrial dysfunction in the WT physiological milieu and that the decreased mitochondrial activity in the dwarf is a consequence of mitochondrial functional plasticity in a prolongevity physiological environment. Our data thus suggest that the decreased CI and CIV activities of the dwarf tissues may be due to hormonal deficiencies and that their mitochondria are not dysfunctional. Thus, although both Ames and WT mice experience these age-associated down regulations of CI and CIV activities, we propose that these mitochondrial changes are specific for WT (ROS) and dwarf (decreased levels of oxidative stress). On the other hand, our data also show that the rate of decline of CI and CIV activities is steeper in the dwarf muscles, which raise the question of whether the hormonal deficiency determines the levels of complex activities, *i.e.,* in aged WT vs. dwarf mice. These observations thus raise the question of whether the decreased CI and CIV activities are characteristic of mitochondrial dysfunction in the WT mice, and plasticity of mitochondrial function in response to the physiological environment caused by the hormonal deficiency of the Ames mouse and that the plasticity may be a prolongevity characteristic.

The decreased expression of certain ETC genes is an evolutionarily conserved mode of lifespan extension in nematodes, flies and mice [16, 49]. Our studies clearly show that decreased expression of CIV activity is more pronounced in the dwarf muscle than in WT muscle tissues, thus suggesting that its level of expression may be a tissue-specific characteristic of cardiac and skeletal muscle in lifespan extension. However, CIV activity does not change in the dwarf kidney. Instead it is the CI, CII, CIII and CV activities that are significantly higher in the dwarf kidney compared to WT. These observations suggest that the longevity-associated activities of the CI-CV complexes and the elevated CI - CIII and CII - CIII activities are tissue-specific, and that lifespan determination in the tissues of complex organisms may involve maintenance of levels of activities of the ETC complexes whose functions reflect the metabolic needs of that particular tissue.

Our studies have shown that there are significant age-related differences between the ETC complex activities from heart, skeletal muscle and kidney tissues in both WT and Ames mice. The differences are more pronounced in the kidney, a slowly proliferating tissue, than in the heart and skeletal muscle, which are post-mitotic tissues. Interestingly, the dramatic decrease in CIV function appears to be tissue-specific for skeletal and heart muscle, which we propose may be a causative factor in leading to decreased energy production in the post-mitotic muscle tissue. On the other hand, the data for the kidney, a slowly proliferating tissue, show improved ETC function, which suggest less of an effect of aging on kidney tissue dysfunction and contribute to extended lifespan of the dwarf mutant. Thus, these altered mitochondrial ETC functions may be the consequence of metabolic changes leading to differences in stress-response pathways and may play a key role in increased resistance to oxidative stress and extended lifespan in the Ames mice. Therefore, our study provides important insights into the physiological effects of the hormonal deficiencies of the Ames mouse, i.e., GH, TSH, PRL, on mitochondrial ETC function.

## METHODS

### Animals and tissues

The Ames colony is maintained at the University of Texas Medical Branch at Galveston. Ames mice were generated by mating *Prop1*^+/-^ heterozygous males and females; progeny were weaned at 1 month of age and tail DNA collected to genotype *Prop1*^+/+^ (WT), *Prop1*^+/-^ (heterozygous), and *Prop1*^-/-^ (dwarf) mice. RT-PCR was performed in a Bio-Rad iCycler using the manufacturer's protocols (Bio-Rad, Hercules, and CA) to clone a small region of the *Prop1* gene; iCycler software was utilized to analyze the results. Mice were housed separately according to their genotype, and WT and dwarf mice were aged for further use. Young (4-5 months), middle-aged (10-12 months) and old (20-26 months) male WT and dwarf mice were maintained in our animal care facility with a 12h light/dark cycle and fed *ad libitum* on a standard chow diet before sacrifice, following all regulations of the UTMB Institutional Animal Care and Use Committee.

### Mitochondrial isolation

Micewere sacrificed by decapitation and their tissues harvested immediately, rinsed in ice-cold PBS, and prepared for mitochondrial isolation. Mitochondria were prepared from the pooled tissues of 8 young, 8 middle-aged or 8 old WT, and 12 young, 13 middle-aged, or 8 old Ames dwarf male mice. Mitochondrial isolation was performed at 4°C as described [50] with minor modifications [40]. Briefly, tissues were blended in a Brinkman Polytron PT 3000 (large blade) for 10-15 seconds in isolation buffer (250 mM sucrose, 0.5 mM EGTA, 2 mM EDTA, 10 mM HEPES-KOH, pH 7.4) containing Antipain, Chymostatin, Leupeptin and Pepstatin A (final concentration 1 mg each/mL). The blended tissues were homogenized 20X with a Teflon pestle homogenizer, and then centrifuged at 800 X g for 20 minutes. The supernatants were collected in separate tubes and the pellets re-suspended in half a volume of isolation buffer each, and homogenized and centrifuged as before. The supernatants were combined with those from the previous step and centrifuged twice more at 800 X g for 20 minutes. Each time the supernatants were transferred to new tubes and the pellets discarded. The final supernatant was centrifuged at 8000 X g for 20 minutes to pellet mitochondria. The mitochondrial pellets were washed 2X each with half volumes and centrifuged at 8000 X g. The final mitochondrial pellets were re-suspended in minimal volumes of isolation buffer, aliquoted and stored at − 80°C. Fresh aliquots were used for each analysis; sonicated mitochondria were generated with a Branson Model 250 Digital Sonifier (2 cycles, 1.8 seconds each, 6 second total time for each cycle; one minute between each cycle, amplitude 30% pulse time on for 0.3 seconds, pulse time off for 0.7 seconds).

### Enzyme activities

Enzyme activities were assayed at room temperature using a Beckman Coulter DU 530 Spectrophotometer (Beckman Coulter, Brea, CA) as described [37, 43]. Citrate synthase activity was measured at 412 nm (e = 13.6 mM^-1^ cm^-1^) as described [51]. Rotenone-sensitive CI, malonate-sensitive CII, antimycin A-sensitive (AA) CIII, KCN-sensitive CIV, and oligomycin-sensitive CV activities, and CI-III and CII-III coupled activities were assayed as described [40, 52, 53].

All activity results are averages of 4 assays from the pooled samples from WT and dwarf mice at each age group. Citrate synthase assay results from young WT were used to calculate ratios of young to middle-aged and young to old, as well as WT to dwarf mitochondrial protein levels and these ratios were multiplied to normalize the enzyme activities for each age group in WT and dwarf. Statistical significance was calculated using the Student's t-test, with p<0.05 and p<0.001 considered significant and highly significant, respectively.

### Polyacrylamide gel electrophoresis

Blue-native PAGE (BN-PAGE) and SDS-PAGE were carried out by established methods [54] with minor modifications [40, 43, 54]. Briefly, a 5 to 12% acrylamide gradient was used for the first dimension BN-PAGE; imidazole was used as the buffer instead of Bis-Tris, and Criterion 10-20% 2D-well gels (Bio-Rad) were used for the second dimension (SDS-PAGE).

### Immunoblotting

Immunoblot analyses were performed as described [40, 43, 55]. Intact mitochondrial ETC complex bands were visualized using antibodies against CI (NDUFA9 subunit), CII (SDHA subunit), CIII (UQCRFS1 subunit), CIV (COX1), and CV (ATP5A1 subunit; all from Molecular Probes, Eugene, OR). Antibody to the mitochondrially encoded COX1 subunit was used as a CIV-specific antibody. All other complex-specific antibodies are against nuclear encoded subunits.
